# Effect of Newborn Resuscitation Training on Health Worker Practices in Pumwani Hospital, Kenya

**DOI:** 10.1371/journal.pone.0001599

**Published:** 2008-02-13

**Authors:** Newton Opiyo, Fred Were, Fridah Govedi, Greg Fegan, Aggrey Wasunna, Mike English

**Affiliations:** 1 Kenya Medical Research Institute (KEMRI)/Wellcome Trust Research Programme, Nairobi, Kenya; 2 Department of Paediatrics and Child Health, College of Health Sciences, University of Nairobi, Nairobi, Kenya; 3 Pumwani Maternity Hospital, Nairobi, Kenya; 4 Department of Epidemiology and Population Health, London School of Hygiene and Tropical Medicine, London, United Kingdom; 5 Department of Paediatrics, University of Oxford, Oxford, United Kingdom; Institute of Clinical Effectiveness and Health Policy, Argentina

## Abstract

**Background:**

Birth asphyxia kills 0.7 to 1.6 million newborns a year globally with 99% of deaths in developing countries. Effective newborn resuscitation could reduce this burden of disease but the training of health-care providers in low income settings is often outdated. Our aim was to determine if a simple one day newborn resuscitation training (NRT) alters health worker resuscitation practices in a public hospital setting in Kenya.

**Methods/Principal Findings:**

We conducted a randomised, controlled trial with health workers receiving early training with NRT (n = 28) or late training (the control group, n = 55). The training was adapted locally from the approach of the UK Resuscitation Council. The primary outcome was the proportion of appropriate initial resuscitation steps with the frequency of inappropriate practices as a secondary outcome. Data were collected on 97 and 115 resuscitation episodes over 7 weeks after early training in the intervention and control groups respectively. Trained providers demonstrated a higher proportion of adequate initial resuscitation steps compared to the control group (trained 66% vs control 27%; risk ratio 2.45, [95% CI 1.75–3.42], p<0.001, adjusted for clustering). In addition, there was a statistically significant reduction in the frequency of inappropriate and potentially harmful practices per resuscitation in the trained group (trained 0.53 vs control 0.92; mean difference 0.40, [95% CI 0.13–0.66], p = 0.004).

**Conclusions/Significance:**

Implementation of a simple, one day newborn resuscitation training can be followed immediately by significant improvement in health workers' practices. However, evidence of the effects on long term performance or clinical outcomes can only be established by larger cluster randomised trials.

**Trial Registration:**

Controlled-Trials.com ISRCTN92218092

## Introduction

Birth asphyxia is estimated to cause 0·7 to 1·6 million deaths a year globally with 99% of these deaths occurring in developing countries [1]. Effective resuscitation could prevent some of these deaths as well as improve the outcomes of surviving asphyxiated babies [1]. However, provision of appropriate newborn resuscitation care is dependent on the presence of an adequately skilled health worker in the home or the facility. To date little attention has been paid to furnishing health workers with these skills and we have little idea what works. We do however know that inappropriate, ineffective or dangerous forms of practice are widespread [2], [3], [4].

In higher income settings Newborn Life Support (NLS) training courses have proliferated. Although these can be expensive little is known about the effect they actually have on health worker behaviour in practice settings [5]. Where studies on the effect of life support training for any age group have been done they focus mostly on knowledge and skill retention observed in simulated practice following course participation. Few studies have examined outcomes considered more useful such as morbidity, mortality or work-place provider practices [5]. Furthermore, the few studies on provider behaviour were all methodologically weak and therefore very little confidence could be attached to their results [5]. The aim of this study was therefore to determine if a simple, one day newborn resuscitation training alters health worker resuscitation practices in a busy public hospital in a low-income setting.

## Methods

The protocol for this trial and supporting CONSORT checklist are available as supporting information; see Checklist S1 and Protocol S1.

### Participants and Randomisation Procedure

The study was conducted in Pumwani Maternity Hospital in Nairobi, Kenya. This is the main maternity facility for Nairobi and provides delivery care to 17,000 women each year. The hospital has approximately 90 nurse/midwives (60 assigned to the labour ward and 30 to the theatre) primarily responsible for delivery care and newborn resuscitation with 14 on duty at any one time (8 labour ward, 6 theatre). A 150 bed newborn nursery, supervised by two paediatricians, provides care for all infants requiring medical attention after delivery. The labour ward has 8 cubicles where deliveries are conducted with resuscitations being performed on one resuscitaire, located no more than 10 metres from the furthest room. The theatre has 2 operating rooms each with a resuscitaire.

Our intention was to test resuscitation training on practices by randomly assigning labour ward and theatre staff to either early or late training, considering the health worker as a unit of clustering. Potential participants, the 90 nurse / midwifery staff, were therefore initially listed by place of work. Eligibility criteria for initial randomisation were: personal work plans for the 3 months post-randomisation that neither included leave of >2 weeks duration, nor rotation to another work station; routine responsibility for newborn resuscitation; provision of informed consent. We aimed to ensure an equal proportion of staff (35%) from labour ward and theatre were included in the early training as this could accommodate at most 32 participants. Those not included in the early training were trained after the initial 3 months observation period.

### Intervention

The intervention was purposely designed by the investigators together with representatives of the Kenya Resuscitation Council under the umbrella of the Kenya Paediatric Association. The form of training drew heavily on the one day UK Resuscitation Council training [6] in form but was significantly adapted to the Kenyan setting where resources are limited. The one day course teaches an A (Airway), B (Breathing) and C (Circulation) approach to resuscitation laying down a clear step by step strategy for the first minutes of resuscitation at birth. It comprises focused lectures aimed at understanding the modern approach to resuscitation and practical scenario sessions using infant manikins to develop skills in airway opening, use of a bag-valve-mask device and chest (cardiac) compressions. Candidates were provided with a simple instruction manual two weeks before the training for self-learning. At the end of the day trainees were assessed using a multiple-choice examination and a formal test scenario evaluating actual practical skills and their integration into a clinical context. Course instructors had completed a Kenya Resuscitation Council Advanced Life Support Generic Instructor Course (GIC) co-supervised by an experienced team from the UK resuscitation council.

### Outcome Measures

The primary outcome for the study was the proportion of resuscitation episodes in which appropriate initial resuscitation steps were practiced as recommended in the NLS training. The primary outcome was further classified into two levels: perfect (where the health worker entirely followed the training guideline) and adequate resuscitation with minor, clinically insignificant deviations from the training guideline (see Appendix S1). The primary steps in recommended resuscitation include only the practices of: suction, restricted only to babies born through meconium yet to take a breath, drying (stimulating), airway examination (A) and positioning and assessment of breathing (B). These practices should occur within the first sixty seconds of any resuscitation making rapid assessment of correct practice possible for an observer. After this actions should depend on whether breathing and subsequently an adequate heart rate are detected, information not necessarily available to an observer. We therefore concentrated on the very early steps as our primary outcome because they should be universal, are readily observable and are objective. In addition, if any problem is identified and the health worker calls for help then for ethical reasons the observers were instructed to provide whatever help they could, under instruction of the primary provider, only recording the step by step actions / instructions of the health worker as soon as possible thereafter. Secondary outcomes were: the frequency of inappropriate and/or potentially harmful practices which might confer a direct risk to the baby or an indirect risk through the delayed initiation of appropriate interventions (see Appendix S2); an overall score awarded to each resuscitation episode after independent review of the documented process by two NLS instructors blinded to the identity or training status of the health worker.

To capture data, trained observers worked a shift pattern to ensure at least one was present in the hospital continuously (spanning all 24 hours) until the estimated number of observations required by our sample size calculations were achieved. When two observers were available (approximately 30% of shifts) one remained on labour ward and one in theatre. When one observer was present they were assigned to either labour ward or theatre by one of the investigators (NO) who was aware of the training allocation to ensure that an adequate number of observations could be collected from each trained health worker. Resuscitation observers were nursing students who had been specially trained as a group over 3 days to make structured observations on newborn resuscitation using role play and scenarios and a standardised checklist. They were not trained in newborn life support. The observers were blind to the training status of the health workers and were instructed not to try to ascertain health workers' training status after discussing with them the possible biases this might introduce and their role in producing a valid research result.

The practice observation check list was based on the resuscitation steps included in the training. Data on events preceding the resuscitation episode, the health workers record of the baby's APGAR score, the availability of equipment and the outcome of the resuscitation were also recorded. All health workers were assigned a unique study code that was the only identifier used on all observation forms.

Routine data on delivery outcomes, admissions to nursery and their causes and outcomes were collected retrospectively for the 6 months prior to the first training (June 2006), for a period of 3 months between the first training and training of the remaining staff (September 2006) and for 3 months after this. We refer to the period between early and late training allowing comparison of practices in trained and untrained providers as phase 1 of the study. In addition, we aimed to observe 50 consecutive resuscitation episodes after the late training to describe practices after ‘saturation training’, this period is referred to as phase 2 of the study.

### Sample-Size Calculations

Our sample size calculation took into account the clustered nature of our data, i.e. resuscitations by the same health worker. Based on routine hospital practice we estimated at best that 3 to 5 observations could be made per health worker over a 6 to 7 week period, a period we reasoned was short enough to reduce the possible effects of cross-group contamination. However, as the proportion of resuscitation episodes that could successfully be observed was unknown we allocated a total period of 3 months for phase 1 observations in case it was required. In the absence of prior data we assumed resuscitation practices were appropriate on average on 50% (standard deviation ±7·5%) of occasions. Further assuming an intra-class correlation coefficient (ICC) of 0.15, [7] a two-tailed test at the 5% significance level and 90% power, we estimated that a minimum of 22 health workers in each arm would need to be monitored with 4 observations made on each (i.e. at least 88 resuscitation events in both intervention and control groups) to detect a 25% absolute change in our primary outcome measure (a 50% improvement) [8]. As these assumptions were based on limited data, particularly with regard to the frequency of our primary outcome and the value of the intra-class correlation coefficient we aimed to train at least 28 health workers in the first training and observe practices for these and for as many of the untrained providers as possible within the practice observation period.

### Data Analysis

All observation checklist data were double entered using MS Access and verified prior to analysis using STATA v.9.2 (Stata Corp., Texas, USA). Two investigators and NRT instructors (ME and FW), blinded to the health workers' identity or training status, independently assigned a score to each resuscitation episode based on review of all of the information on the observation sheet and using a 5 point scale, where 5 represented perfect resuscitation (see Appendix S3). Scores were compared and individual cases where scores differed by >1 point were discussed by the two investigators with a revised, agreed final score applied. For cases where scores differed by ≤1 point the average of the two scores was considered the final score.

Observations were linked by the unique health worker study code and all analyses accounted for non-independence. Our analysis took into consideration the clustered nature of data in that health workers cared for more that one neonate. We used a cluster adjusted chi-square test to compare the proportions of appropriate initial resuscitation steps between the intervention and control groups. For the frequency of inappropriate practices and to compare the mean score for resuscitation performance we used a cluster adjusted two sample *t* test. We report risk ratios (RR) and 95% confidence intervals (CIs) (also adjusted for clustering) for the primary outcome. Confounding was explored for the categorical variables sex, years of experience (categorised as ≥median or <median) and place of work (labour ward or theatre) by calculating stratified, cluster adjusted risk ratios. After adjusting for these potential confounders there was no clinical or statistically significant variation in the main outcome of interest.

### Ethics

The study was conducted with the permission of the hospital management to whom we explained the implications, purpose and voluntary nature of participation. Similar information was made available in written form to all labour ward and theatre staff and written informed consent was obtained from all health workers prior to their practice being observed. Information on the nature and purpose of the study and the need for the presence of an observer was also given to mothers admitted to the hospital for delivery. Mothers were given the opportunity to decline the presence of a resuscitation observer. As this hospital serves a national population of almost 3 million people we did not attempt to gain ‘community consent’ outside the hospital. Ethical approval for the conduct of the study was obtained from the Kenya Medical Research Institute / National Ethics Committee.

## Results

**Figure 1:**
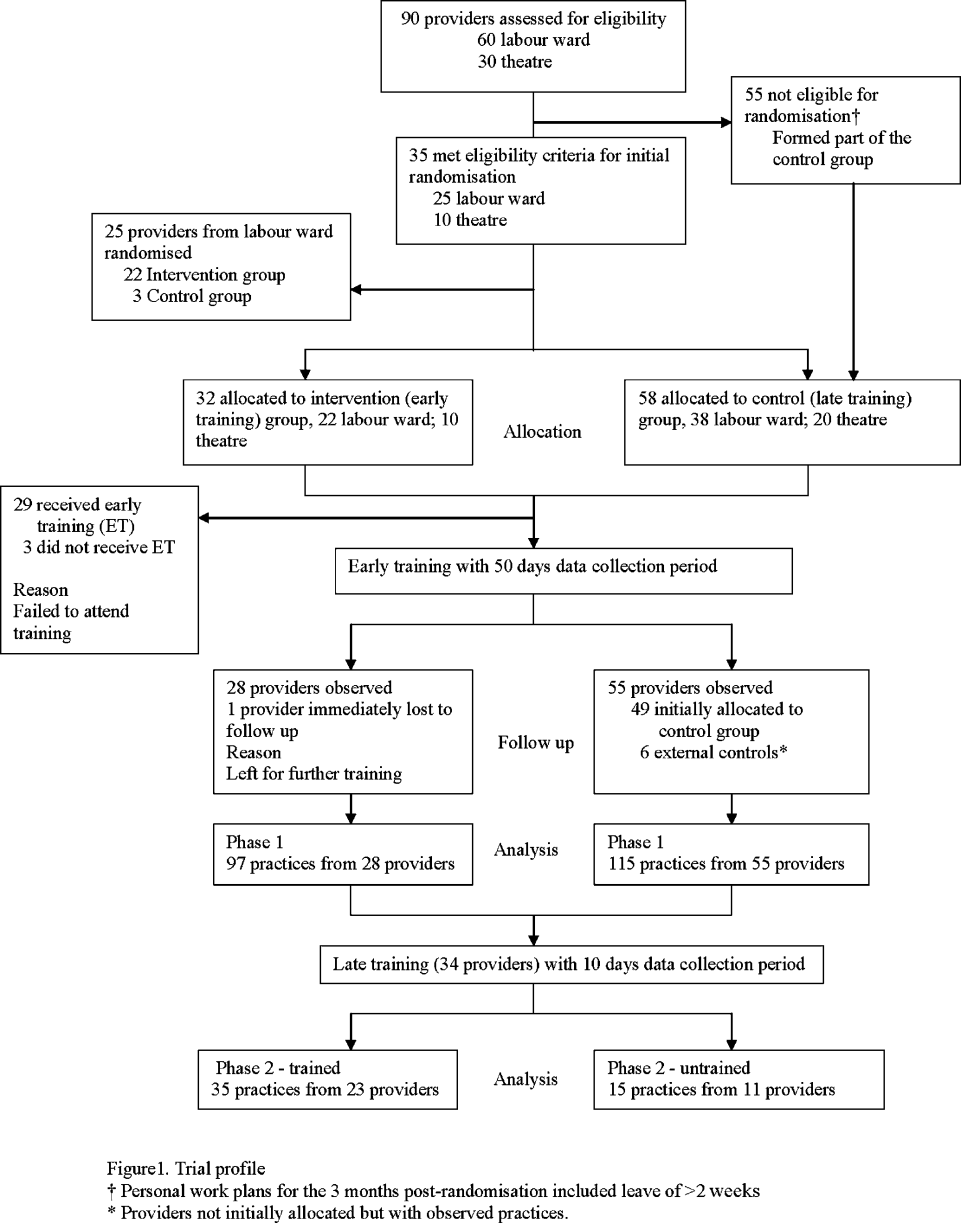
Trial profile

Although our intention was to randomise staff, stratified by place of work (labour ward or theatre), to early or late training this proved to be impossible for the most part as a large number of potentially eligible staff did not meet our inclusion criteria because of expected absences of >2 weeks in the 3 months observation period for leave, scheduled off-duty periods or attendance at training seminars (figure 1). The final allocation of participants and process of observation is summarised in figure 1. Most of the providers were females (trained; females 89.3 % (25/28), males 10.7 % (3/28), untrained; females 78.2% (43/55), males 21.8 % (12/55). There were no significant differences in the ages (median age (interquartile range, IQR); trained, 36(27–47), untrained, 35(27–51) and years of experience between the groups with the majority of health workers being relatively junior (median years worked (IQR), trained 1(1–20), untrained 1(1–20). Two hundred and twelve resuscitation episodes were observed for 83 providers in phase 1 while 50 were from 34 providers in phase 2. Ninety seven of the phase 1 practices were from 28 trained providers while 115 were from 55 untrained providers. Thirty five of the phase 2 practices were from 23 trained providers while 15 were from 11 remaining untrained providers. The profile of study patients and nursery admissions and deaths is summarised in table 1.

**Table 1:** Profile of study patients

	Pre-intervention phase		Post-intervention phase	
	Period 1	Period 2	Period 3	Period 4
Number of deliveries	4367	4302	4205	4084
Stillbirths				
Fresh	67	80	69	54
Macerated	64	66	60	62
Neonatal deaths†	7	9	5	7
Birthweights				
<2000 g	213	223	194	211
2000–2499 g	362	339	286	312
2500–4000 g	3668	3663	3667	3629
>4000 g	102	72	70	82
Illness specific nursery admissions and deaths				
Birth asphyxia				
<2000 g	66(13)	35(8)	21(7)	19(3)
2000–2499 g	75(23)	92(10)	54(1)	51(5)
2500–4000 g	474(23)	495(17)	441(20)	426(38)
>4000 g	23(0)	17(0)	16(1)	9(0)
Prematurity	152(37)	165(34)	137(42)	197(39)
RDS (Term)	44(0)	48(6)	37(7)	80(12)
RDS (Preterm)	12(5)	11(3)	12(5)	8(2)
Neonatal sepsis	40(0)	46(1)	19(0)	35(1)
Jaundice	25(0)	33(2)	26(1)	11(0)
MAS	0(0)	0(0)	2(0)	6(0)
Congenital abnormality	14(1)	16(1)	17(1)	21(0)
Neonatal mortality rate* (95% CI)	25.0 (20.5–30.0)	21.2 (17.1–25.9)	21.4 (17.3–26.2)	26.2 (21.5–31.6)

† Deaths during resuscitation;

* In-hospital rate per 1000 live births

RDS: respiratory distress syndrome; MAS: meconium aspiration syndrome

Deaths are given in parentheses

For our primary outcome in phase 1, we observed a significantly higher proportion of perfect initial resuscitation steps (24%) among trained providers compared to the control group (10%) (Risk ratio [RR] 2.27, 95% CI 1.23–4.22; p = 0.009, adjusted for clustering) (Table 2). Similarly, the proportion of adequate initial resuscitation steps was higher among trained (66%) providers as compared to the control group (27%) (RR 2.45, 95% CI 1.75–3.42; p<0.001, adjusted for clustering). Analyses taking account of a possible confounding effect of the baseline imbalance in gender did not alter the observed effect of training; adequate resuscitation, RR 2.34, 95% CI 1.67–3.27, p<0.001, adjusted for sex and clustering). Results from analyses based on pooled data from both phase 1 and 2 periods were similar (Table 2). Risk ratios calculated for individual time periods each representing one third of the follow-up time in phase 1 did not demonstrate any converging trend (data not shown), arguing against a significant effect of contamination, although clearly there was limited power to detect anything but a major effect.

**Table 2:** Group comparison for appropriate initial resuscitation steps (all analyses are cluster adjusted)

	Mean	Risk ratio (95% CI)	p-value
**Phase 1**			
Perfect resuscitation	23.7%/10.4%	2.27 (1.23–4.22)	0.009
Adequate resuscitation	66.0%/27.0%	2.45 (1.75–3.42)	<0.001
**Phase 2**			
Perfect resuscitation	40.0%/13.3%	3.00 (0.79–11.42)	0.064
Adequate resuscitation	74.3%/60.0%	1.24 (0.71–2.15)	0.312
**Phase 1 and 2**			
Perfect resuscitation	28.0%/10.8%	2.60 (1.53–4.43)	<0.001
Adequate resuscitation	68.1%/30.8%	2.22 (1.64–2.99)	<0.001

Similarly comparisons of trained and untrained providers for phase 1 and phase 1 and 2 combined showed significantly fewer inappropriate and potentially harmful practices (summarised in Appendix S2) per resuscitation in the trained group (Phase 1: Mean difference 0.40 (trained 0.53 vs untrained 0.92), 95% CI 0.13–0.66; p = 0.0038) (Table 3). A total of 256 (98.0%) resuscitation episodes were documented sufficiently well to permit scoring. Phase 1 group comparison showed significantly higher average resuscitation scores in the trained group as compared to the control group (Mean score: trained 2.50, 95% CI 2.25–2.74; untrained 1.95, 1.74–2.16, p = 0.0008). This effect was also apparent using pooled data from Phase 1 and 2 (Table 3). In consecutive observations in the period after late training the proportion of resuscitation episodes with adequate initiation of resuscitation was 70% (95% CI 51.4%–88.7%).

**Table 3:** Mean number of inappropriate/harmful practices and resuscitation scores per episode (all analyses are cluster adjusted)

	N	Clusters	Mean (95% CI	p-value
**a) Inappropriate and dangerous practices**				
**Phase 1**				
Intra-cluster correlation = 0.20				
Untrained = 0	115	55	0.92 (0.75–1.10)	
Trained = 1	97	28	0.53 (0.32–0.73)	
Difference (0–1)	212	83	0.39 (0.13–0.66)	0.0038
**Phase 1 and 2**				
Intra-cluster correlation = 0.19				
Untrained = 0	130	61	0.87 (0.72–1.02)	
Trained = 1	132	51	0.45 (0.29–0.61)	
Difference (0–1)	262	112	0.42 (0.21–0.64)	0.0002
**b) Mean resuscitation scores**				
**Phase 1**				
Intra-cluster correlation = 0·12				
Untrained = 0	112	54	1.95 (1.74–2.16)	
Trained = 1	94	28	2.50 (2.25–2.74)	
Difference (0–1)	206	82	−0.55 (−0.86, −0.23)	0.0008
**Phase 1 and 2**				
Intra-cluster correlation = 0·12				
Untrained = 0	127	60	1.83 (1.61–2.04)	
Trained = 1	129	51	2.40 (2.18–2.61)	
Difference (0–1)	256	111	−0.57 (−0.87, −0.27)	0.0003

Group comparison for the overall mortality in all the resuscitation episodes showed no statistically significant differences between the groups (Trained 0.28 (18/65), 95% CI 0.17–0.40; control 0.25 (9/25), 0.12–0.42, p = 0.77). Additionally, no significant differences were seen in birth asphyxia admission and fatality rates before and after training (Table 1). For birth asphyxia pre-intervention admission rates to the newborn unit among infants weighing 2000–4000g were 13.1% of all births, 95% CI 12.4%–13.8% while post-intervention they were 11.7%, 11.0%–12.4%. Fatality rate amongst infants weighing >2000g admitted to the newborn unit with asphyxia was 6.4%, (5.1%–8.0%) in the pre-intervention period and 6.6% (5.1%–8.3%) in the period following late training.

## Discussion

We attempted to undertake a cluster-randomised trial to study the effect of a simple one day newborn resuscitation training on health worker practices. However, our criteria for randomisation, aiming to ensure health workers were present to be observed in a defined period, resulted in few staff being eligible. We cannot therefore discount the possibility of bias in group allocation although we feel this is unlikely. The training intervention significantly improved the performance of initial resuscitation steps, with 66% initial practices being adequate in the intervention group compared with 27% in the control group. In addition, there were significant reductions in the frequency of inappropriate and potentially harmful practices and improvements in overall resuscitation scores. There was no obvious effect of training on mortality of babies resuscitated, no obvious decline in asphyxia admission rates and no overall decline in newborn mortality in the hospital as the number of trained providers increased. However, this study was neither specifically designed nor powered with mortality as the primary outcome and our mortality results are best used to inform the design of future studies. In addition, appropriate initial resuscitation is clearly only the first stage in a continuum of effective care, not addressed by this intervention, that is likely to be required to prevent many adverse outcomes from severe asphyxia.

We are not aware of any previous randomised controlled studies examining the effect of resuscitation training on provider practices in a true clinical setting. The majority of studies on newborn resuscitation have focussed on less direct outcomes such as participants' knowledge and skills [5], [9], [10]. Such surrogate outcomes may not necessarily reflect practice changes, a more useful and direct way of measuring the effectiveness of resuscitation training programmes [5]. Although our primary study outcome was only able to capture the initial steps in effective practice we believe it does indicate an important behaviour change effect, especially if considered together with the reduction in unnecessary / potentially harmful practices and an improvement in overall resuscitation scores.

Previous studies and our control group data demonstrate that both resuscitation skills and knowledge are poor despite frequent exposure to situations in which both are needed [3], [11] Internationally, there is now considerable consensus on how newborn resuscitation should be provided [12] and it is believed that in 95% cases when it is required resuscitation should be possible with only a minimum of equipment and without access to intensive care skills or facilities [4], [13]. Recent research findings have strengthened this opinion demonstrating that suction in the presence of meconium and the use of oxygen are in most newborns unnecessary [14], [15], [16], [17]. These findings have relevance to our study as the failure to provide suction to a non-breathing baby born through meconium as the first step was a major reason for failing to achieve a ‘perfect’ classification in our primary outcome. If, as seems likely, there is little value of suction in these babies then a substantial clinical impact from our intervention, 66% of adequate appropriate practices in trained providers, might be a more reasonable interpretation than the modest impact suggested by only 25% of initial practices in trained providers being perfect.

Our data add to a body of knowledge suggesting some improvement in clinical outcomes [10], [18] or in acquisition of knowledge and skills of providers following resuscitation training [9]. In a systematic review on the effectiveness of all types of life support courses all the three mortality and morbidity studies indicated a positive impact, with an overall odds ratio of 0.28 (95% CI 0.22–0.37). However, no net increase in scores in 5/8 studies of retention of knowledge and in 8/9 studies of skills retention were apparent, although all the studies assessing behavioral outcomes were reported to be methodologically weak [5].

Similarly, our study has limitations. Attempts to randomise health workers had limited success. We cannot exclude the possibility of cross-group contamination, although this would tend to reduce the apparent effect of the intervention. In contrast it is likely that the difficulty in maintaining observer blinding could bias the results in favour of an intervention effect. If the observers, even unintentionally, were more likely to view the practices of a provider they came to know was trained as correct this would bias our results despite our efforts in training to limit this effect. We also only observed practitioners for a short period after training and are unable to provide any information on the duration of the training effect. In the few studies assessing the duration of effect a rapid and linear decay in cardio-pulmonary (CPR) skills -from as early as two weeks after training, with skills deteriorating to pre-training levels by one year, have been reported [11], [19], [20], [21].

For low-income countries Life Support Courses are associated with relatively high direct and opportunity costs (learners'/instructors' time, equipment purchase, etc). While there is increasing pressure to implement such courses it is important that their true effects on actual health worker performance and ideally morbidity and mortality are established. Such studies need to be based in typical, low-income settings where supervision and opportunities for continuous learning or ongoing mentorship and resources for post-resuscitation care may be limited. In addition, they should perhaps consider a range of possible training delivery mechanisms, be embedded in local health systems to promote sustainability, assess impact over the long term and consider costs and cost effectiveness to optimise appropriate health policy decisions. Clearly such studies will require appropriate levels of funding.

In conclusion, our findings suggest that implementation of a simple one day newborn resuscitation training can be followed by significant, short-term improvement in health workers' practices. To ensure a high proportion of all resuscitation episodes are appropriately managed clearly a large majority of providers must be trained. Evidence on effects on long term performance or clinical outcomes, however, remain inconclusive and can only be established by larger trials. The availability, accessibility and correct functioning of basic resuscitation equipment is still a missing essential pre-requisite for the success of training and resuscitation itself in many settings [2].

## Supporting Information

Checklist S1 CONSORT Checklist
(0.25 MB PDF)

Protocol S1 Trial Protocol
(0.06 MB PDF)

Appendix S1 Levels of appropriate initial resuscitation steps
(0.04 MB RTF)

Appendix S2 Inappropriate and harmful practices
(0.04 MB RTF)

Appendix S3 Scoring instrument for the assessment of resuscitation practices
(0.04 MB RTF)
