# Supplementary material for: Effect of Newborn Resuscitation Training on Health Worker Practices in Pumwani Hospital, Kenya
Source: PLoS One. 2008 Feb 13;3(2):e1599. doi: 10.1371/journal.pone.0001599 (PMC2229665; doi:10.1371/journal.pone.0001599)
Supplement: Appendix S1 — Levels of appropriate initial resuscitation steps (0.04 MB RTF) [file pone.0001599.s003.rtf]

Appendix S1. Levels of appropriate initial resuscitation steps
Outcome	Definition	
Perfect   resuscitation¶	Followed training guideline without deviation
     No meconium: Dry  → Check/Open Airway → Check breathing (B)
     Meconium present, baby floppy: Suction →  Dry  → Check/Open 
    Airway → Check breathing (B) 
	
Adequate  resuscitation¶	Minor deviations from training guideline
     No meconium: Dry  → Check/Open Airway → Check breathing (B)
     Meconium present, baby floppy:  Dry  → ± Suction → Check/Open 
    Airway → ± Suction → Check breathing (B)
	
Incorrect resuscitation	Deviations from training guideline and not classified above¶	
