# Supplementary material for: Effect of Newborn Resuscitation Training on Health Worker Practices in Pumwani Hospital, Kenya
Source: PLoS One. 2008 Feb 13;3(2):e1599. doi: 10.1371/journal.pone.0001599 (PMC2229665; doi:10.1371/journal.pone.0001599)
Supplement: Appendix S2 — Inappropriate and harmful practices (0.04 MB RTF) [file pone.0001599.s004.rtf]

Appendix S2. Inappropriate and harmful practices
