# Supplementary material for: Effect of Newborn Resuscitation Training on Health Worker Practices in Pumwani Hospital, Kenya
Source: PLoS One. 2008 Feb 13;3(2):e1599. doi: 10.1371/journal.pone.0001599 (PMC2229665; doi:10.1371/journal.pone.0001599)
Supplement: Appendix S3 — Scoring instrument for the assessment of resuscitation practices (0.04 MB RTF) [file pone.0001599.s005.rtf]

Appendix S3. Scoring instrument for the assessment of resuscitation practices
Score	Interpretation	
5	Resuscitation entirely appropriate- no inappropriate practices
	
4	Resuscitation good- no inappropriate practices, minor, clinically insignificant deviations from recommended sequence
	
3	Resuscitation adequate and not dangerous- inappropriate practices do not interfere with care or threaten outcome, deviations from recommended sequences unlikely to be of significance
	
2	Resuscitation poor- inappropriate practices or deviations from recommended sequences could have some impact on outcome (significant delay in establishing adequate ventilation, potential for mild adverse effect)
	
1	Resuscitation very poor and  potentially dangerous- inappropriate practices or deviations from recommended sequences significantly delay effective care in a very sick baby or can cause possibly serious adverse consequences 
	
