# Supplementary material for: Effect of Newborn Resuscitation Training on Health Worker Practices in Pumwani Hospital, Kenya
Source: PLoS One. 2008 Feb 13;3(2):e1599. doi: 10.1371/journal.pone.0001599 (PMC2229665; doi:10.1371/journal.pone.0001599)
Supplement: Protocol S1 — Trial Protocol (0.06 MB PDF) [file pone.0001599.s002.pdf]

1) An operational evaluation of introducing training in newborn resuscitation for maternity ward staff at Pumwani Hospital.

2) INVESTIGATORS.

Principal Investigator: Dr. M English, 1*

Co- investigators: Opiyo Newton, 1*

Dr. F Govedi, 2 ¶

Dr. F Were, 3 ¶

Dr. S Nesbitt, 4 ¶

Prof. A. Wasunna, 3 ¶

1) Centre for Geographic Medicine Research – Coast, KEMRI / Wellcome Trust Collaboration, P.O. Box 230, Kilifi, Kenya. (* and P.O. Box 43640, Nairobi)
2) Consultant Paediatrician, Pumwani Hospital, Nairobi.
3) Department of Paediatrics and Child Health, College of Health Sciences, University of Nairobi, Nairobi.
4) Gertude's Garden Children's Hospital, Muthaiga, Nairobi.

¶ CVs supplied in Appendix.

3) ABSTRACT:

Birth asphyxia is estimated to result in 0.7 to 1.6 million deaths a year globally with 99 % of these deaths occurring in developing countries. While improved management of labour could reduce this burden of disease effective newborn resuscitation is also required. Personnel in Kenya’s public health facilities however receive little training in modern approaches to newborn resuscitation. We intend to assess the degree to which a simple training approach based on the best available evidence is able to change health workers’ practices when providing newborn resuscitation care in a large public hospital. Before and after the intervention we will also monitor the outcome of deliveries at the hospital, focusing on the morbidity and mortality of term infants, the group most likely to benefit from the delivery of more effective care.

4) INTRODUCTION:

Birth asphyxia is estimated to result in 0.7 to 1.6 million deaths a year globally with 99 % of these deaths occurring in developing countries [1,2] Overall deaths in the first 7 days of life account for 23 % neonatal mortality, with prematurity adding to the burden attributable to asphyxia [2]. In Kenya the practical impact of such statistics is revealed in one study in a district hospital maternity department where death before discharge occurred in 1 of 33 babies born alive [3]. Prematurity and birth asphyxia were the main causes of these deaths [3].

To prevent some of these deaths it has recently been argued that mothers should be strongly encouraged to deliver in health facilities where health professionals can provide essential care for mother and baby [4]. This major shift in thinking has occurred as a result of the apparent failure of two decades of efforts to reduce mortality and morbidity by promoting the use of traditional birth attendants [4-7]. The critical assumption, however, is that health facilities and their staff are able and equipped to provide effective interventions.

In Kenya at present there is little formal training of health staff (nurses, nurse/midwives, clinical officers or doctors) in resuscitation of the newborn. In fact in many hospitals even the most basic equipment for such resuscitation is lacking [8]. This is likely to result in many cases in situations where health staff provide inappropriate, ineffective or even dangerous forms of care. For example it is not uncommon for the administration of glucose via an umbilical venous catheter to be considered a priority by many staff in newborn resuscitation even before the establishment of a secure airway and adequate ventilation (personal observations of the investigators).

Internationally there is, however, now a reasonable degree of consensus on how newborn resuscitation should be provided and it is widely believed that for 95% babies resuscitation should be possible with only a minimum of equipment and without access to intensive care skills or facilities [9]. Recent research findings have strengthened this opinion demonstrating that access to effective suction equipment is not routinely required for either suction of the oropharynx of a baby on the perineum or for suction of the trachea in a vigorous baby born through meconium stained liquor [10-12]. Similarly it is now appreciated that oxygen is not required for most episodes of newborn resuscitation, with air

perhaps even having some advantages over resuscitation with oxygen [13-17]. Therefore even in resource constrained environments the costs of providing effective newborn resuscitation to the majority of babies who need it should not be high.

The aim of the proposed study is therefore to determine if, and to what degree, specific training in newborn resuscitation results in a change in practice of health care staff in a busy public hospital. The training will be adapted from that provided by the European Resuscitation Council [18] according to local facilities and needs and is anticipated to occupy one full day. The impact of the training intervention on admission rates to the special care nursery in the hospital or on survival of term infants, the group likely to benefit most from effective resuscitation in a low income country setting, will also be monitored.

5) JUSTIFICATION.

Improving newborn survival presents a major challenge in Kenya. As strategies include delivery of high-risk infants in health facilities it is vital that staff at these facilities have the necessary skills and equipment to provide effective newborn resuscitation. We aim to test the ability of a standard training course, adapted to Kenya's context, to promote the provision of effective, evidence based practices in a public hospital setting and to examine the possible impact of such training on outcomes of care.

6) NULL HYPOTHESIS:

Provision of training in newborn resuscitation does not change health workers' practices when providing routine care.

7) OBJECTIVES.

General Objectives.

To examine the ability of training to improve newborn resuscitation practices and to explore possible impact on delivery outcomes for term babies using a 'before and after' study design.

Specific Objectives.

1) To examine the ability of training to change health workers' practices when providing newborn

resuscitation using a cluster randomized trial design.

2) To describe the outcomes of delivery before and after provision of training in a large public hospital. The primary outcomes to be monitored are rates of admission to the special care nursery of term babies with asphyxia and their survival.

8) DESIGN AND METHODOLOGY.

a) Study Site.

The study will be presented to the senior medical and administrative staff responsible for Pumwani Maternity Hospital in Nairobi and their approval sought before the study begins. This hospital provides delivery care to 25,000 women each year and is the main maternity facility for the city of Nairobi. The hospital has approximately 90 nurse / midwives (60 assigned to the labour ward and 30 to the theatre) primarily responsible for delivery care and newborn resuscitation with 14 on duty at any one time (8 labour ward, 6 theatre). A 150 bed newborn nursery, supervised by two paediatricians, is also provided where all infants requiring care after delivery are referred. In the months of June to August 2005 the newborn nursery received approximately 400 admissions each month with 60% associated with asphyxia (F. Govedi, unpublished data).

b) Study Population.

The study proposed is in two parts. A cluster randomised trial of training will be undertaken to assess the impact of training on health worker practices. An observational study of the outcomes of delivery will be undertaken before, during and after the training intervention.

<u>The Cluster Randomised Trial.</u>

All nurse/midwives expected to provide delivery care and newborn resuscitation for a period of at least 3 months after the start of the trial will be eligible for inclusion as subjects of the trail. A list of these staff will be made (anticipated number 75) and 40% will be randomly selected and will comprise the early training group. The remainder will comprise the control or late training group and will be trained after completion of trial data collection (see below, estimated to take 1-2 months). Staff will be asked to consent to this process and to have their practice observed (see below).

Observational Survey of Outcomes.

For a period of 3 months before the cluster randomised trial data will be collected on the outcomes

of all deliveries. Particular attention will be paid to the collection of high quality data on the number of term infants delivered, the number of term infants admitted to the newborn nursery and the number of newborn infants dying and the cause of these deaths. Collection of this data will continue during the trial and for a 3 months period after those in the late training group have been trained.

c) Sampling.

i) Sample size:

The Cluster Randomised Trial.

Observations of the practice of newborn resuscitation are clearly not independent events – they are linked to the health worker responsible for the resuscitation. While the primary data collection (see below) will therefore be on episodes of resuscitation the primary unit of analysis (and randomisation) must therefore be the health worker. There are few data to inform sample size calculations of this sort. However, if we assume that resuscitation practices are appropriate on average on 50% (SD +/- 7.5%) of occasions now (a generous assumption) and that training could improve this proportion to 75%, and assuming an average intraclass correlation co-efficient of 0.15, a two-tailed test and power 90%, then the null hypothesis could be safely rejected if observations on four resuscitation episodes were collected from twenty-two health workers receiving early training and twenty two health workers who had not yet received training (a control group) [19]. Thus, data would be collected from at least 88 (4 x 22) resuscitation events in both the intervention and control groups (a total of at least 176 events).

As these assumptions are based on little data – particularly with regard to the proportion of outcome prevalence and the intraclass correlation co-efficient – we intend to collect data on up to 5 resuscitation episodes for twenty eight health workers in the training group and more in the control group that should ensure our ability to detect differences of at least the magnitude described.

Observational Survey of Outcomes.

At present Pumwani Maternity Hospital provides delivery care for over 2,000 women per month. Of these 1,600 are term babies and 200 per month or 10% are admitted with 'asphyxia' with 30 deaths per month representing 15% term asphyxiated admissions (F. Govedi, unpublished data)). Confidence limits around the relevant proportions after 3 months observation would thus be: admission rate – 9.25% to 10.8%, case fatality 12% to 18%. A reduction in absolute admission rate of 2% or relative reduction of 20% might therefore be greater than one would expect by chance and

plausibly related to the intervention if these differences were observed in the pre-intervention period compared with the period after the control group have received training. Similarly a reduction in case fatality rate to below 10% might be greater than one would expect by chance and plausibly related to the intervention.

ii) Sampling procedure

The Cluster Randomised Trial.

The selection of health workers for early or late training has been discussed above. As early as possible after training, to reduce cross-group contamination, at least 3 and a maximum of 5 episodes in which a health worker has to provide resuscitation will be observed and data recorded on a specific proforma.

Observational Survey of Outcomes.

Routine data from ward registers (maternity and nursery) will be collected by a research assistant to determine: the number of deliveries, the outcomes of delivery (including whether a term or preterm baby, stillbirth or early death), the numbers of admissions to the nursery and their causes and outcomes. All data will be collected from routine hospital sources (eg. ward registers) and will not involve questioning of the mother or family.

d) Procedures.

The training intervention will be supervised by trainers who have completed the European Resuscitation Council's Advanced Life Support Generic Instructor Course. The training will last 7 working hours and will be delivered on site. This training comprises focused lectures aimed at understanding the modern approach to resuscitation and practical training using infant manikins to develop skills in airway opening, use of a bag-valve-mask device to inflate the chest and chest (cardiac) compressions. The course teaches an A (airway), B (breathing), C (circulation) approach to resuscitation laying down a clear, step by step strategy for the first minutes of resuscitation for all resuscitation episodes conducted by nurse / midwives. It is possible to train up to 32 candidates at one time if at least 9 instructors are available. Course materials will be provided to selected candidates 2 weeks before the training for self-learning.

i) Data collection.

Routine data collection of births, outcomes and admissions has been described.

Women admitted to the Pumwani hospital for delivery will be informed at the time of their admission

that research is being conducted to assess the skills of health workers in providing assistance to babies who have problems breathing at birth. Women will be offered the opportunity to say they do not wish the birth of their baby and its immediate post-delivery care to be observed as a consequence of the study of health workers' skills. Thus women will be given an opportunity to opt out of any association with the study.

For observations of resuscitation episodes research assistants who are themselves health workers or health workers in training will be trained to record the process of resuscitation on a standard proforma. The proforma will focus on whether the resuscitation proceeds appropriately through the first two steps recommended in training to open the airway (A) and provide effective ventilation with 5 rescue breaths with at least 2 resulting in chest expansion (B). Any deviation from this A and B initial approach will be regarded as inappropriate. Other resuscitation practices will be observed and recorded but the proportion of occasions with appropriate performance of steps A and B will be the primary outcome of the cluster randomised study. The observers will not be told whether the health worker has been trained or not (although maintaining complete blinding in this type of study is unlikely to be possible) and accuracy of objective recording will be emphasised during the observers' training. It will not be possible to protect against a Hawthorne effect in this study but the presence of such an effect is as likely to improve performance in the control group as in the intervention group and will make it harder to reject the null hypothesis.

ii) Data validation.

Resuscitation observers will be carefully trained together so that their recording of events is consistent within and between observers. A study data supervisor (ON) will monitor the quality of the data being collected on a daily basis and periodically provide a second observation of resuscitation as part of an ongoing data quality assurance process.

Routine sources of hospital data will be cross-referenced for accuracy.

9) DATA MANAGEMENT.

Data will be collected using observation and data abstraction forms and subsequently entered into custom developed databases using Filemaker Pro in a PC format. Range and consistency checks will be built into the initial data entry. All databases will be collated and cleaned prior to analysis and final

copies kept with the principal investigator on a PC with back-up copies on CDs. Individual level data will not include names or area of residence and all records will be indexed on and labelled by unique study record identifiers only. Only summary data and not individual level data will be provided to the hospitals in the form of feedback reports.

Data on the performance of health workers' resuscitation practices will be handled sensitively with reports never identifying an individual health worker. However, as the interests of patients are also at stake poorly performing health workers will be offered further training where necessary although there will be no punitive action.

Data Analysis

Data analysis will be conducted using Stata v 8.0 software (Stata Corp., Texas, USA) and the proportion of episodes of appropriate initial resuscitation compared between intervention and control groups using analyses appropriate to the clustered data. For the description of newborn outcomes, including admission rates, simple proportions, ratios and confidence intervals will be calculated from the routinely collected data. As there are a potentially large number of confounders that could affect admission and mortality rates, many of which will be hard to account for, conclusions about the impact of training on these outcomes will be made with considerable caution.

10) TIME FRAME:

Baseline data collection	-	January & February 2006
Conduct of cluster randomised trial	-	March – May 2006.
Completion of training and continued observational data collection	-	June – August 2006
Analysis and report preparation	-	September – December 2006.

11) ETHICAL CONSIDERATIONS:

- The study tackles a problem of major public health importance in Kenya and aims to improve health worker knowledge and practice.
- It is important to be able to demonstrate the value of training in newborn resuscitation so that it can be more widely promoted if it is valuable, or, if it proves to have little effect, so that alternative methods of changing practice can be explored.
- Health workers will be fully informed about the study and that their practice will be observed and recorded. However, they will also be informed that their performance data will be kept confidentially and will not be used in any punitive way, the effort being to help them improve.
- Observers will be trained carefully to preserve the confidentiality of the health worker and also of the mother and baby during observations.
- Observers are primarily responsible for data collection but where their assistance is required to support the health worker in caring for either a very sick mother or very sick baby then this will over-ride any other obligation.
- Although mothers of infants delivered in the hospital are not the object of the study and will not be approached for formal consent the purpose of the study and the need for the presence of an observer will be explained to them early on in their admission. At this stage or at any point up to the time of their infant's birth they will be able to decline such observation.
- It is possible that in a few instances a direct benefit of having an observer present will be gained as staff shortages often mean health workers lack immediately available help in emergency situations.
- It is hoped that babies of mothers delivering at the hospital will benefit in the future from

improvements in staff skills.

Animal Subjects.

Not applicable

12) EXPECTED APPLICATION OF THE RESULTS.

The results are expected to provide important information on newborn resuscitation practices in Kenya's largest maternity hospital and on one possible means to improve them in line with modern evidence. Should a positive benefit be observed then this will strengthen the case for introducing training in newborn resuscitation more widely in Kenya's health facilities and training institutions.

13) REFERENCES.

1. Lawn J, Cousens S, Zupan J, Team LNSS. 4 million neonatal deaths:When?Where?Why? *Lancet* 2005;**365:**891 –900.
2. WHO. The World Health Report 1995. Bridging the gap. 1995.
3. English M, Muhoho A, Aluda M, Were S, Ross A, Peshu N. Outcome of delivery and cause-specific mortality and severe morbidity in early infancy: a Kenyan district hospital birth cohort. *Am J Trop Med Hyg.* 2003;**69**(2)**:**228-232.
4. WHO. The World Health Report 2005. Make every mother and child count. 2005.
5. Raina N, Kumar V. Management of birth asphyxia by traditional birth attendants. *World Health Forum* 1989**:**243-246.
6. Goodburn E, Chowdhury M, Gazi R, Marshall T, Graham W. Training traditional birth attendants in clean delivery does not prevent postpartum infection. *Health policy and planning* 2000**:**394-399.
7. Sibley L, Sipe T. What can a meta-analysis tell us about traditional birth attendant training and pregnancy outcomes? *Midwifery* 2004;**20:**51-60.
8. English M, Esamai F, Wasunna A, Were F, Ogutu B, Wamai A, Snow R, Peshu N. Delivery of paediatric care at the first-referral level in Kenya. *Lancet* 2004;**364:**1622-1629.
9. WHO, Manh, S. Basic newborn resuscitation: a practical guide. 1997.
10. Wiswell T, CM G, Jacob J, Goldsmith L, Szyld E, Weiss K, Schutzman D, Cleary G, Filipov P, Kurlat I, Caballero C, Abassi S, Sprague D, Oltorf C, Padula M. Delivery room management of the apparently vigorous meconium-stained neonate: Results of the multicenter, international collaborative trial. *Pediatrics* 2000;**105:**1-7.
11. Halliday H, Sweet D. Endotracheal intubation at birth for preventing morbidity and mortality in vigorous, meconium-stained infants born at term (Review). The Cochrane Database of Systematic Reviews, 2001.
12. Vain N, Szyld E, Prudent L, Wiswell T, Aguilar A, Vivas N. Oropharyngeal and nasopharyngeal suctioning of meconium-stained neonates before delivery of their shoulders:Multicentre,randomised controlled trial. *Lancet* 2004;**364:**597-602.
13. Saugstad O, Rootwelt T, Aalen O. Resuscitation of asphyxiated newborn infants with room air or oxygen: An international controlled trial: The resair 2 study. *Pediatrics* 1998;**102**(1).
14. Vento M, Asensi M, Sastre J, Garcýa-Sala F, Pallardo F, Vina J. Resuscitation with room air instead of 100% oxygen prevents oxidative stress in moderately asphyxiated term neonates. *Pediatrics* 2001;**107:**642-647.
15. Ramji S, Rasaily R, Mishra P, Narang A, Jayam S, Kapoor A, Kambo I, Mathur A, Saxena N, Saxena B. Resuscitation of asphyxiated newborns with room air or 100% oxygen at birth: A multicentric clinical trial. *Indian Pediatrics* 2003;**40:**510-517.
16. Vento M, Asensi M, Sastre J, Lloret A, Garcia-Sala F, Vina J. Oxidative stress in asphyxiated term infants resuscitated with 100% oxygen. *J Pediatr.* 2003;**142**(3)**:**240-246.
17. Tan A, Schulze A, O'Donnell C, Davis P. Air versus oxygen for resuscitation of infants at birth (Review): The Cochrane Database of Systematic Review, 2004.
18. Phillips B, Zideman D, Wyllie J, Richmond S, van Reempts P. European resuscitation council guidelines 2000 for newly born life support :A statement from the paediatric life support working group and approved by the executive committee of the european resuscitation council. *Resuscitation* 2001;**48:**235-239.
19. Hayes RJ, Bennett S. Simple sample size calculation for cluster-randomized trials. *Int J*

Epidemiology 1999;**28:**319-326.

14) BUDGET.

Personnel & Training	KSH	$
Research Assistant, Senior Nurse, 12 months	930,000	12,400
Resuscitation Observers	200,000	2,666
Training of Pumwani nurses (2 courses for 28 participants)	80,000	1,066
**Equipment & consumables**		
Resuscitation training equipment	75,000	1,000
Computer	90,000	1,200
Office consumables	60,000	800
**Total**	**1,435,000**	**19,133**

* This assumes an exchange rate of 75 KSH / 1 USD

These costs will form part of the KEMRI / Wellcome Trust collaborative programme based in Kilifi and Nairobi and as such will not incur additional overhead costs.

15) JUSTIFICATION OF THE BUDGET.

A senior nurse will be required to collect the hospital data on outcomes, to organise the newborn resuscitation training, to train observers and to collect and enter their data. Four observers will be required for two months to permit 24 hour monitoring of resuscitation practices. To support the study a desktop computer will be required and basic office costs (that include occasional transport to the study site) will be supported. To facilitate the training and potentially to provide an opportunity for self-training some basic resuscitation training equipment (infant manikins, airway and breathing support devices) will be purchased that will remain the property of the hospital at the end of the study.

Appendix 1 – Consent.

A study to examine the effect of training on newborn resuscitation practices

Consent Information Sheet for health workers.

About KEMRI

KEMRI is part of the Ministry of Health and runs research activities to find out more about illness and how to manage illness in Kenya. The overall aim is to improve health and well being for people in Kenya and other parts of Africa.

In the research at this hospital KEMRI is working with other Kenyan groups including senior staff at this hospital and The University of Nairobi.

What is this research activity?
We would like to find out whether a simple one day training on the modern, recommended approach to resuscitate newborn babies that need help to breathe immediately after birth actually helps people practice in a better way. At the same time we would like to see, if the training does work, whether there is any change in the number of babies that die or the number who need care on the nursery.

Who are we approaching?
We are approaching all the health workers who might be expected, routinely, to provide resuscitation to a newborn infant during the course of their normal work .

What are we asking people to do?
We are asking two main things:

1) *We wish first to train one group of staff and after a short period during which we will observe how babies are resuscitated we will train the next group(s) of staff. We will not choose who gets trained first and who gets trained later deliberately. We will use a method that selects people by chance. We ask you to agree to be trained and to agree to this method of deciding when the training will be done.*
2) *To know whether the training makes a difference to the way people practice we will need to observe episodes when health workers are actually resuscitating babies and make a record of this. This will mean an observer being present when a health worker is taking care of a baby. We ask you to agree to being observed in this way.*

Confidentiality.
Your name and job title will not be used in any reports of this work. Only a code number will appear on the record made of the observation(s) and only the research team will have access to the link between the code numbers and individuals. No one other than the research team will be allowed to see the record of the observation(s) without your permission. We will not use any of the records to report on an individual worker's performance. We will only report how groups perform as a whole.

Risks of the research.

We do not believe there are any risks to taking part in this research.

Benefits of the research

We are unable to offer any individual benefits for participating in this research other than the receipt of a short training course. However it is hoped that this research will help to improve the care given to newborn babies in the hospital.

Voluntary Participation.

There is no obligation at all to help with this study and there will be no penalties of any kind if you decide not to take part. If you do agree to start helping with the study at any time you may change your mind and ask not to be involved any further.

Do you have any questions?

Consent Agreement for Health Workers for for study to examine the effect of training in newborn resuscitation on health worker practices.

I, ___________________________________ have been informed about the study to examine the effect of training on newborn resuscitation practices under the direction of Dr. M. English and have been provided with information concerning this study to help me understand it. The implications, duration, purpose, voluntary nature and inconveniences or risks that may reasonably be expected have been explained to me by:

___________________________ *(name of person taking consent).*

I have been given the opportunity to ask questions concerning the study and these have been answered to my satisfaction. If I have further questions, I may contact:

Dr. M. English
P.O. Box 43640
Tel.2720163
Nairobi.

I understand that I may at any time during the study revoke my consent without any loss or penalty and that the information I have contributed will then be destroyed.

I confirm that I:

1) Am happy to be involved and have my practice observed by the study team

Signed:___________________________ Date ____ / ____ / _________

Signature of person taking consent

____

A study to examine the effect of training on newborn resuscitation practices

Information for mothers admitted for delivery.

<u>About KEMRI</u>

KEMRI is part of the Ministry of Health and runs research activities to find out more about illness and how to manage illness in Kenya. The overall aim is to improve health and well being for people in Kenya and other parts of Africa.

In the research at this hospital KEMRI is working with other Kenyan groups including senior staff at this hospital and The University of Nairobi.

<u>What is this research activity?</u>
We would like to find out whether training staff how to help babies with problems breathing immediately after birth actually helps them practice in a better way and whether babies might benefit, for example by needing care in the nursery less often.

Who are we studying?
We are studying all the nurses who might need to help babies breathe by watching how they give this help to babies. To watch what the nurses do a member of the study team will need to watch how the nurse cares for your baby as soon as it is born.

<u>What are we asking from you?</u>
We would like to make sure you are happy for someone who is not the nurse caring for you to attend your baby's birth and watch the nursing care he/she receives. The study personnel are themselves health workers.

<u>Confidentiality.</u>
Your name and / or your babies name will not be recorded or used at all – it is the hospital's nurse we are studying.

<u>Benefits of the research</u>
We are unable to offer any individual benefits but it is hoped that this research will help to improve the care given to newborn babies in this hospital.

<u>Saying no.</u>
There is no obligation at all to help with this study and if you would not like a study team member to watch how the health worker gives care to your baby you are free to say so - there will be no penalty of any kind.

If you have any questions please ask a member of the staff or the study team.

If you do not want the study team member to be present when your baby is born please tell the nurse taking care of you.

Appendix 2. CVs.

Name: Prof. Aggrey Wasunna,

Dob 4th August 1953

Current Address; Department of Paediatrics and Child Health,
Faculty of Medicine,
College of Health Sciences,
University of Nairobi,
P.O. Box 19676, Nairobi.

Qualifications:	1978	MBChB (Distinctions: Pathology, Paediatrics, Community Medicine, Credits: Biochemistry, Physiology, Obstetrics/Gyanecology, Internal Medicine, Surgery)
	1981	Certificate in Tropical Medicine.
	1984	M.Med (Paediatrics). Best overall results.
	1985	Specialist recognition, Paediatrics.
	1986-88	Fellow, Neonatal Medicine and Registrar in Paediatrics – Royal Postgraduate Medical School, Hammersmith, University of London.
	1989	Sub-specialist recognition, Neonatal & Developmental Medicine.

Current Position: Associate Professor of Neonatal Medicine / Paediatrics and Chairman, Department of Paediatrics and Child Health, University of Nairobi.
Consultant Neonatologist/Paediatrician, Kenyatta National Hospital,
Chief of Neonatal Services, Agha Khan Hospital, Nairobi.

Recent Activities	2002	Member of the International Scientific Association of Probiotics and Prebiotics (involved in the development of studies involving these products in collaboration with The Lawson Institute, Canada.)
	2000	First International Hepatitis B Advisory Board Meeting, Atlanta, USA.

Current Publications:

Wasunna, A.O. and Mohammed, K. Low birthweight babies: Some sociodemographic and obstetric characteristics of adolescent mothers at the Kenyatta National Hospital Nairobi.

Wasunna, A.O. and Mohammed, K. Morbidity and outcome of low birthweight babies of adolescent mothers at the Kenyatta National Hospital, Nairobi.

Wasunna, A.O. and Dubowitz, L. Protective head turning response in preterm infants: Possible effects of theophylline administration and intracranial abnormalities.

Name: Frederick N. Were.

Dob. 30th August 1958.

Current Address; Department of Paediatrics and Child Health,
Faculty of Medicine,
College of Health Sciences,
University of Nairobi,
P.O. Box 19676, Nairobi.

Qualifications:
1984 MBChB, University of Nairobi.
1988 Certificate in Tropical Medicine.
1990 M.Med (Paediatrics). Best overall results.
1993 Fellow In Neonatal ?Perinatal Medicine at Monash Medical Centre, Monash University, Melbourne, Australia.
1994 Specialist recognition, Paediatrics.
1998 Sub-specialist recognition, Neonatal Physician.

Current Position
Lecturer in Neonatal Medicine / Paediatrics and coordinator of Postgraduate studies in the Department of Paediatrics.
Consultant Neonatologist at Kenyatta National Hosp ital.
Chairman of the Medical Advisory Committee at the Mater Hospital.
Member of the Medical Advisory Committee, Gertrude's Children's Hospital.

Other activities
Chairman of the Scientific Committee of the Kenya Paediatric Association since 1995.
Clinical Educator for the implementation of the Pentavalent Vaccines introduced in East Africa from 2001.

Current publications.

Were, F.N and Mukhwana, R.O. Neonatal outcomes of babies born less than 2000 grams at Kenyatta National Hospital, Kenya.

Were, F.N and Mukhwana, R.O Early perinatal mortality in Kenyatta National Hospital, Nairobi, Kenya.

Were, F.N. and Ndigwa, D.N. Hyponatraemia in sick Very Low Birthweight Infants.

Name: Fridah A Govedi.

Current Address; Consultant Paediatrician,
Pumwani Hospital,
Nairobi.

Qualifications:
1995 MBChB, University of Nairobi.
2002 M.Med (Paediatrics).

Current Position
Consultant Paediatrician and Paediatrician in Charge of the Newborn Unit, Pumwani Hospital, Nairobi, 2002 to 2005.

Other activities
Member of Kenya Paediatric Association.

CURRICULUM VITAE DR. SIDNEY JAMES NESBITT

PROFESSIONAL REGISTRATION AND MEMBERSHIP DETAILS

1. Paediatric Specialist Recognition (KMP&DB) April 2000
2. Registration (KMP&DB) 19 February 1996 A No: 003856

ACADEMIC HISTORY:

November 1994 Royal College of Physicians (U.K). **MRCP (U.K.) Paediatrics**
1983-1989 Trinity College, Dublin, Ireland **B. A. MB B.A.O. B.Ch**

Academic Presentations/Papers

1. KPA Scientific Conference (Mombasa - August 2003)	Update on Paediatric Poisoning
2. SA Paediatric Conference (Durban, Sept 2001)	Evidence Based Paediatrics Made Easy
3. KPA Scientific Conference (Mombasa - April 1998)	2 Cases of *Kawasaki Disease* diagnosed at - **The *Royal Free Hospital, London***
4. Journal of the Royal Society of Medicine (July 1994, Vol. 87)	Yersinia Pseudotuberculosis in a 3 year old boy with rapid rapid response to Cefotaxime
5. The Royal Society of Medicine (London - February 1993)	Use of *Centoxin* (Monoclonal Antibody) in Meningococcal Septicaemia **Presentation**
6. The Royal Society of Medicine (London - November 1992)	*Yersinia Pseudotuberculosis* in a 3 year old boy with rapid response to Cefotaxime - **The *Royal Free Hospital, London***

Postgraduate Continuous Medical Education Courses

1.	June 2000	Instructor on PALS Courses- **Addenbookes Hospital, Cambridge & Kings College Hospital, London, UK**
2.	June 2000	Study day on **Long Term ventilation in children**. *The Hospital for Sick Children, Great Ormond, London.*
3.	September 1999	**PALS (**Paediatric Advanced Life Support) **Instructors Course** – *Churchill/John Radcliffe Hospital, Oxford, U.K.*
4.	May 1999	Update on **Paediatric Respiratory Diseases** – *The Hospital for Sick Children, Great Ormond, London*
5.	December 1998	**PALS (**Paediatric Advanced Life Support) - *Kings College Hospital, London*
6.	July 1997	**APLS** (Advanced Paediatric Life Support) - *Nairobi Hospital*

POST GRADUATE EXPERIENCE

PAEDIATRIC CLINIC, GERTRUDE'S GARDEN CHILDREN'S HOSPITAL (GGCH)
July 1998 – Present date:
Current Clinical and Administrative responsibilities:
1. **2000-2005** Selected to be a member of the **Board of Trustees** of GGCH
2. 2000-2005 member of the **Medical Advisory Committee** of GGCH
3. 2005 Selected to be chairman of the **Education Department** at GGCH by the Medical Advisory Committee (see attached summary of GGCH academic activities)
4. **1999 – 2005** Director of the GGCH **Resuscitation Department**.
(see attached summary of GGCH academic activities)
5. **2004- 2005** Director of **Paediatric HIV Training Programme** at GGCH.
(see attached summary of GGCH academic activities)
6. **2001- 2005** Clinical Representative on the Gertrude's Garden Children's Hospital **Computerisation Committee**.
7. **2002-2005** **Honorary Lecturer** Department of Paediatrics, University of Nairobi.

8. **2001- 2005** Secretary to the **Kenya Paediatric Association** (KPA)
